# Treatment-related adrenal insufficiency in patients with autoimmune blistering diseases

**DOI:** 10.1016/j.jdin.2024.11.001

**Published:** 2024-11-24

**Authors:** Chloe Choy Heng Yoong, Lydia Tang-Lin, Jielin Yew, Chia Chun Ang

**Affiliations:** a Department of Dermatology, Changi General Hospital, Singapore; b Department of Endocrinology, Changi General Hospital, Singapore; c Department of Dermatology, Singapore General Hospital, Singapore

**Keywords:** adrenal, autoimmune blistering diseases, cortisol, HPA axis, hypothalamic pituitary axis, insufficiency, pemphigoid, pemphigus, prednisolone, prednisone, side effects, steroid

*To the Editor:* Systemic corticosteroids are the backbone in the treatment of autoimmune blistering diseases, but its use is associated with metabolic comorbidities including adrenal insufficiency.^1^ The prevalence of treatment related adrenal insufficiency in autoimmune blistering diseases patients is rarely reported.^2^^,^^3^

We routinely assessed autoimmune blistering diseases patients for adrenal insufficiency when they are in clinical remission on ≤5 mg prednisolone, prior to treatment cessation (Fig 1). Patients who declined testing were monitored for adrenal insufficiency clinically during the prednisolone taper. We conducted an audit of those patients seen between Jan 2010 and September 2023 in Changi General Hospital who underwent a short Synacthen test. Cases were identified from the hospital’s electronic database using ICD codes for bullous pemphigoid, pemphigoid, pemphigus, pemphigus vulgaris, and pemphigus foliaceus. We excluded patients who had an existing diagnosis of adrenal insufficiency or had missing data regarding the prednisolone treatment doses and duration. Masked demographic and treatment data were extracted in those cases who underwent the short Synacthen test. Data were analyzed using nonparametric Wilcoxon Rank Sum test with SPSS (version 29.0.2.0) (IBM).

**Fig 1:**
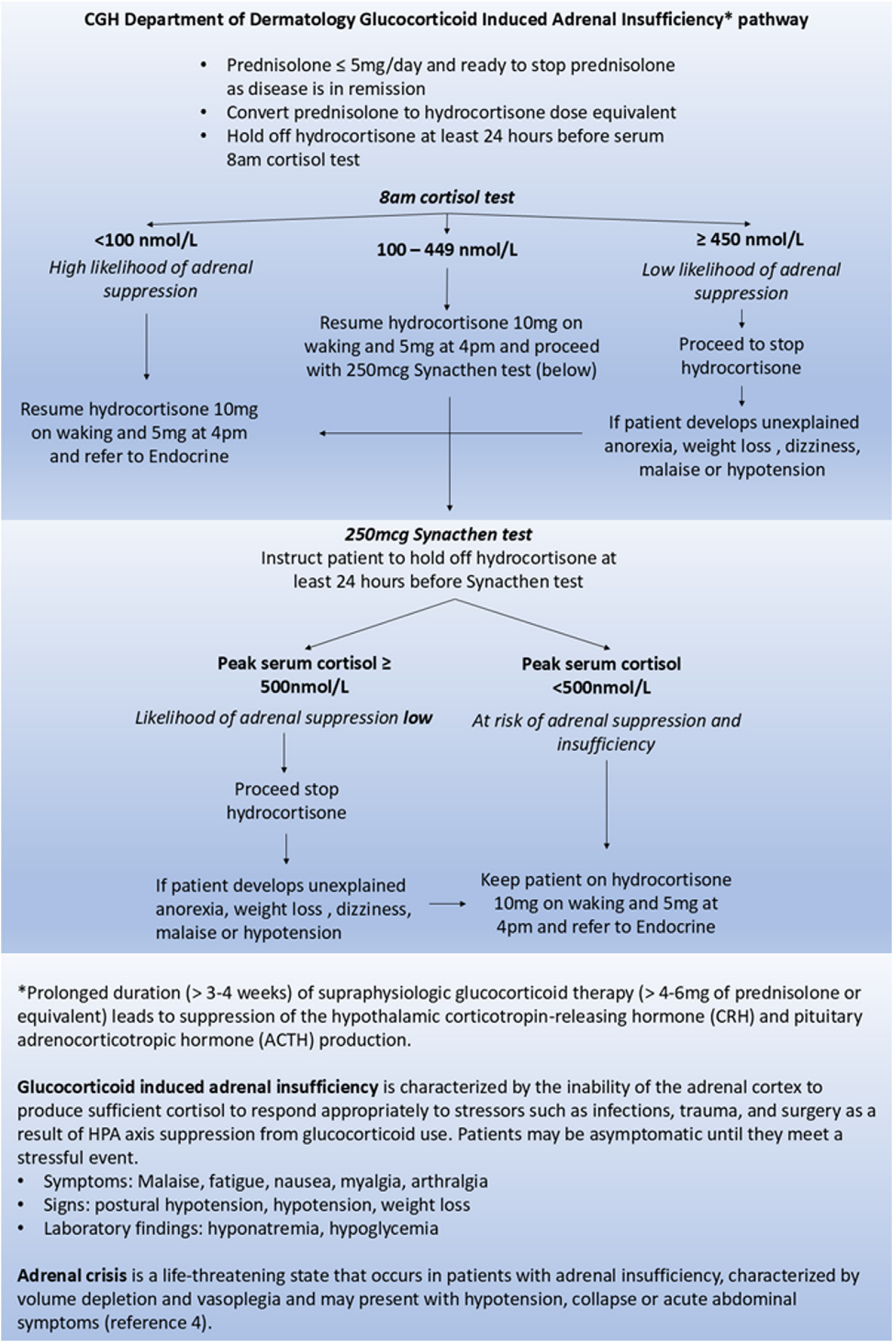
Definitions of adrenal insufficiency and adrenal crisis, and CGH Department workflow for evaluation of adrenal insufficiency in patients who were previously on systemic corticosteroids.

Twenty-three patients were included (Table I). Bullous pemphigoid accounted for 65% of the cohort (15 patients). The median age was 78 years. The median duration of their skin disease was 48 months. The median initiating dose of prednisolone was 20 mg and the median cumulative prednisolone dose was 5502 mg. Eighteen patients (78%) also required the use of steroid-sparing agents.

**Table I:** Baseline characteristics of AIBD patients who underwent Synacthen test for adrenal insufficiency (AI)

	Total (*n* = 23)	Patients without AI (*n* = 15)	Patients with AI (*n* = 8)
Age (y), median (range)	78 (54-98)	75 (54-98)	83 (63-91)
Gender, *n* (%)			
Male	13 (57)	8 (53)	5 (63)
Female	10 (43)	7 (47)	3 (37)
Ethnicity, *n* (%)			
Chinese	19 (83)	14 (93)	5 (63)
Malay	3 (13)	1 (7)	2 (25)
Indian	1 (4)	0 (0)	1 (12)
Comorbidities			
Metabolic syndrome (DM, HTN, HLD)	15 (65)	9 (60)	6 (75)
Asthma	2 (7)	2 (13)	0
Autoimmune conditions	0	0	0
Rheumatologic conditions	0	0	0
Diagnosis, *n* (%)			
Bullous pemphigoid	15 (65)	9 (60)	6 (75)
Pemphigus foliaceus	3 (13)	3 (20)	0
Pemphigus vulgaris	4 (18)	2 (13)	2 (25)
Anti Laminin gamma 1 pemphigoid	1 (4)	1 (7)	0
Duration of disease (mo), median (range)	48 (2-156)	53 (19-156)	18 (2-120)
Prednisolone dose (mg), median (range)			
On initiation	20 (10-50)	20 (10-50)	17.5 (10-40)
Cumulative prescribed dose	5502 (420-54,950)	5820.5 (1050-54,950)	3650 (420-48,514)
Median duration of prednisolone intake in days (range)	728 (49-4503)	945 (70-4503)	517 (49-2877)
Prednisolone dose (mg/d), (range)∗	7.3 (2.5-19.2)	6.9 (2.5-19.2)	8.3 (4.6-17.3)
Use of steroid-sparing agents, *n* (%)			
Yes†	18 (78)	12 (80)	6 (75)
Doxycycline and/or Nicotinamide	14	9	5
Dapsone/Azathioprine/Mycophenolate mofetil/Rituximab/Cyclosporine/IVIG	8	5	2
No	5 (22)	3 (20)	2 (25)

*AIBD*, Autoimmune blistering diseases.

∗ Calculated by first finding the mean prednisolone dose/day (total prescribed dose divided by steroid days) for each patient, then using the median value for this measure for each of the 3 groups analyzed (all patients, those with and those without adrenal insufficiency).

† 8 out of 18 patients had trialed both doxycycline and/or nicotinamide, alongside other biologics/immunosuppressants as part of treatment.

Short Synacthen test was performed electively for 15 patients per department protocol, while 6 patients were tested because of hyponatremia (1 patient), hypotension or incidental postural hypotension (5 patients). The reasons for testing were not available in the electronic records for 2 patients. Eight patients (34%, Table I) were diagnosed with adrenal insufficiency and were managed by the endocrinologist with oral hydrocortisone replacement and periodic assessment for recovery of adrenal function. The median age for this group was 83 years. Six (75%) had bullous pemphigoid and 2 (25%) had pemphigus vulgaris. The median duration of disease was 18 months. The median prednisolone dose on initiation was 17.5 mg, median cumulative prednisolone dose was 3650 mg and median prednisolone dose per day was 8.3 mg/day. There was no statistically significant association between the duration of disease (*P* = .06), duration of prednisolone intake (*P* = .21), cumulative prednisolone dose (*P* = .38), median prednisolone dose (*P* = .59), use of steroid-sparing agents (*P* = .79), and adrenal insufficiency. We were unable to compare our results with another study of adrenal insufficiency in pemphigus patients as a nonstandard measurement of adrenal insufficiency was used in that study.^3^

Our experience highlights the importance of assessing for adrenal insufficiency at the end of systemic steroid therapy,^4^ as up to a third of our patients were affected. This was a small single center audit which was underpowered to detect any statistically significant risk factors and limits generalizability. Future studies should consider the confounding effects of pre-existing adrenal suppression, intake of systemic steroids from other healthcare providers, pace of prednisolone taper and the use of topical steroids over large body surface areas (for example, whole body topical clobetasol in bullous pemphigoid^5^).

## Conflicts of interest

Dr Ang received honoraria from Biogen and Bristol Myers Squibb. Drs Choy Heng Yoong, Tang-Lin, and Yew have no conflict of interests to declare.
